# Correction: The effect of information content on acceptance of cultured meat in a tasting context

**DOI:** 10.1371/journal.pone.0240630

**Published:** 2020-10-07

**Authors:** Nathalie C. M. Rolland, C. Rob Markus, Mark J. Post

There is an error in affiliation 2 for author Nathalie Rolland. The correct affiliation 2 is: Department of Nutrition and Food Science, AgroSup, Université Bourgogne Franche-Comté, Dijon, France.
