# Supplementary material for: The effect of information content on acceptance of cultured meat in a tasting context
Source: PLoS One. 2020 Apr 16;15(4):e0231176. doi: 10.1371/journal.pone.0231176 (PMC7162467; doi:10.1371/journal.pone.0231176)
Supplement: S1 Text — (DOCX) [file pone.0231176.s001.docx]

**Appendix A: online invitation questions**

Welcome to this study!

We invite you to participate in a study about meat.

The study will take place at the Maastricht university….

…

Please fill in the following form.

We will contact you soon to inform you if you are selected for the study.

Please indicate your gender:

- Female
- Male

Please specify your age:

In which city do you live?

Please specify your social economic status:

- Higher managerial, administrative or professional
- Intermediate managerial, administrative or professional
- Supervisory or clerical and junior managerial, administrative or professional
- Skilled manual workers
- Semi-skilled and unskilled manual workers
- Casual or lowest grade workers, pensioners, and others who depend on the welfare state for their income

Do you have food allergies?

- Yes
- No

If yes, to what food?

Do you have dental problems that could prevent you from chewing?

- Yes
- No

Are you taking medicines that could affect your taste, smell and concentration?

- Yes
- No

When are you available to come to the Maastricht University on weekdays:

- Lunch time
- Late evening

Please leave your email address:

Please leave your phone number:

Thank you for your participation.


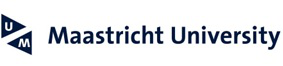
OS

**Appendix B: Online survey prior to visit at intake**

**Study: Appreciation of meat by consumers**

*Welcome to this study of the University of Maastricht. Before you actually participate in the study, please answer the following questions by ticking the appropriate box. Please answer the questions as honestly as possible. All answers are kept confidential, edited and used anonymously. There are no right or wrong answers.*

- 1. How concerned are you about environment and climate change?
- Very much
- fairly
- a bit
- barely
- Not
- Absolutely not
  1. How concerned are you about animal welfare?
- Very much
- fairly
- a bit
- barely
- Not
- Absolutely not
  1. How concerned are you about hunger in the world and food security?
- Very much
- fairly
- a bit
- barely
- Not
- Absolutely not
  1. How concerned are you about food safety?
- Very much
- fairly
- a bit
- barely
- Not
- Absolutely not

**Appendix C: Questionnaire during visit**


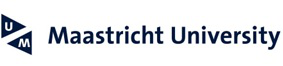
Q1

Ref:

**Study: Appreciation of meat by consumers**

*Welcome to the University of Maastricht.*

*Thank you for participating in the study on the appreciation of meat by consumers. In this study you will be asked questions about your meat consumption. You will also be asked to test two pieces of hamburger, followed by a few more questions. The whole takes about 1 hour.*

*Please answer the questions as honestly as possible. There are no right or wrong answers, answers that best reflect your opinion are the best. All data is treated confidentially and stored anonymously (not directly traceable to personal information).*

*In case of uncertainties, please ask the researcher for clarification.*

1.1) How often do you eat meat (meat, beef, pork, chicken, turkey, game, fish or shellfish)? Also consider snacks, sandwich spreads and meals at home and outdoors.

- Twice or multiple times a day
- Once a day
- 5-6 times a week
- 2-4 times a week
- Once a week
- 2- 3 times a month
- Once a month
- Less than once a month
- never
  1. How often do you eat beef (hamburger, steak, roast beef, ready-made beef)? Also think of snacks, sandwich spreads and meals at home and outdoors
- Twice or multiple times a day
- Once a day
- 5-6 times a week
- 2-4 times a week
- Once a week
- 2- 3 times a month
- Once a month
- Less than once a month
- never

*Please read first:*

Cultured meat is made from cow stem cells. These cells are obtained through a harmless needle biopsy from a muscle. The stem cells are cultured to very high numbers. Once sufficient cells are cultured, they are stimulation to make muscle or fat tissue, the primary components of meat. Muscle and fat tissue are then kneaded into a hamburger patty as we know it.

*Now please answer the following questions by checking the chosen answer (only 1 answer possible):*

- 1. Have you heard about cultured meat?
- No
- Yes, but I do not know what it is
- Yes, and I do know what it is
  1. What is your opinion on cultured meat?
- I am in favor
- I am probably in favor
- do not know
- I am probably against
- I am against
  1. Do you like to try cultured meat?
- definitely
- probably
- I do not know
- probably not
- definitely not
  1. Will you buy cultured meat when it becomes available in the supermarket?
- yes, definitely
- probably
- I do not know
- probably not
- definitely not

- 1. Are you willing to replace your current meat consumption by consumption of cultured meat?
- yes, definitely
- probably
- I do not know
- probably not
- definitely not


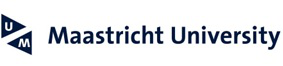
Q2

Ref:

**Study: Appreciation of meat by consumers**

*Please read the following text (subjects get presented with either of the three information boxes, depending on the group they are assigned to): Please read the following information:*

**INFORMATION 1: Group 1: Social benefits**

Meat is delicious, but its production through animal husbandry presents a big problem in the near future. The world's population is growing and the demand for meat increases with increasing prosperity, especially in India and China. This increase in demand cannot be met by livestock farming. In addition, we know that livestock farming is responsible for 15-20% of all greenhouse gas emissions, especially methane from cows. Finally, intensive livestock is associated with animal suffering.

Cultured meat offers a solution to all those problems.

With more efficient use of raw materials for cultured beef production, 90% less land, 90% less water and 60% less energy is needed than for livestock meat. Because we need much fewer cows, greenhouse gas emissions are greatly reduced. Reduction of deforestation, return of farmland to nature, and reduction of the greenhouse effect can mitigate and possibly reverse climate change. Smaller livestock populations mean radical changes in the ways animals are kept; humanely treated populations can be maintained without the use of antibiotics and growth hormones.

Animals do not have to be slaughtered.

With cultured meat you can continue eating meat with a clear conscience.

**INFORMATION 2: Group 2: Personal Benefits**

Cultured beef has the same composition as regular beef and it is therefore completely safe for eating. It is made of the muscle stem cells of a healthy, organically bred cow. That means that cultured beef has the same nutritional value as regular meat. By the controlled culture method, you are assured that beef is not infected with bacteria or diseases such as mad cow disease. In addition, cultured beef does not contain antibiotics or hormones and has not been genetically modified.

By culturing meat, the quality of each piece of meat can be guaranteed and standardized. The fat content and type of fat such as polyunsaturated fatty acids or omega-3 fatty acids can be determined and composed as desired. This would make cultured meat even healthier than the regular meat.

Cultured meat is safe and approved by the Food and Consumer Product Safety Authority.

**INFORMATION 3: Group 3: Quality and Taste**

Eating meat is an experience that is seen as natural and necessary. Cultured meat is the only alternative to plain meat that consists of real meat. It therefore has the same taste, smell, tenderness, juiciness, and mouthfeel as conventional meat. It is also a product that you can make to your personal taste. You can determine how much fat you want in your meat and how it is marbled. Quality of the cultured meat can be guaranteed because production is standardized. Meat specialists select the best and tastiest ingredients to make your favorite meat. Cultured meat can satisfy the demands of all types of consumers, from the gastronome who wants high quality or adventurous meat products to the fast food consumer who goes for easy preparation or ready-to-eat meat.

You can enjoy every cut of meat: your favorite burgers, steak or entrecote, BBQ meat with your family on holidays.

We can offer meat of all kinds of cow breeds, each with their own distinct flavor.

Cultured meat contains all the nutritional components of traditional meat and is an excellent source of protein.

*Please answer the following questions again by checking the chosen answer (only 1 answer possible):*

Q2

- 1. What is your opinion on cultured meat?
- I am in favor
- I am probably in favor
- do not know
- I am probably against
- I am against
  1. Do you like to try cultured meat?
- definitely
- probably
- I do not know
- probably not
- definitely not
  1. Will you buy cultured meat when it becomes available in the supermarket?
- yes, definitely
- probably
- I do not know
- probably not
- definitely not

- 1. Are you willing to replace your current meat consumption by consumption of cultured meat?
- yes, definitely
- probably
- I do not know
- probably not
- definitely not


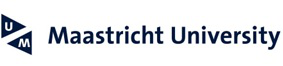
App

Ref:

*Your performance on the tasks you perform during this experiment are partly influenced by your current need for food. That is why we would like to know the following:*

- 1. How hungry are you?

|  |  |  |  |  |  |  |  |  |  |  |
| --- | --- | --- | --- | --- | --- | --- | --- | --- | --- | --- |
| 0 | 1 | 2 | 3 | 4 | 5 | 6 | 7 | 8 | 9 | 10 |
| not at all |  |  |  |  |  |  |  |  |  | very much |

- 1. How do you feel?

|  |  |  |  |  |  |  |  |  |  |  |
| --- | --- | --- | --- | --- | --- | --- | --- | --- | --- | --- |
| 0 | 1 | 2 | 3 | 4 | 5 | 6 | 7 | 8 | 9 | 10 |
| Very bad |  |  |  |  |  |  |  |  |  | excellent |

- 1. How much do you feel like having a meal right now?

|  |  |  |  |  |  |  |  |  |  |  |
| --- | --- | --- | --- | --- | --- | --- | --- | --- | --- | --- |
| 0 | 1 | 2 | 3 | 4 | 5 | 6 | 7 | 8 | 9 | 10 |
| not at all |  |  |  |  |  |  |  |  |  | very much |

- 1. How much do you fancy a savour snack right now?

|  |  |  |  |  |  |  |  |  |  |  |
| --- | --- | --- | --- | --- | --- | --- | --- | --- | --- | --- |
| 0 | 1 | 2 | 3 | 4 | 5 | 6 | 7 | 8 | 9 | 10 |
| not at all |  |  |  |  |  |  |  |  |  | very much |

- 1. How much do you fancy a sweet snack right now?

|  |  |  |  |  |  |  |  |  |  |  |
| --- | --- | --- | --- | --- | --- | --- | --- | --- | --- | --- |
| 0 | 1 | 2 | 3 | 4 | 5 | 6 | 7 | 8 | 9 | 10 |
| not at all |  |  |  |  |  |  |  |  |  | very much |


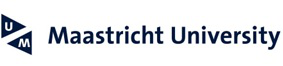
SE

Ref:

**Study: Appreciation of meat by consumers**

*You are about to taste / review 2 pieces of hamburger; a piece of a regular hamburger and a piece of a cultured hamburger. Both hamburgers consist of beef and the cultured meat resembles normal meat as much as possible. Both are also prepared in exactly the same way. The piece of cultured hamburger is smaller because it is not yet produced in large quantities.*

*You must then indicate for each piece of hamburger how you rate it in terms of appearance, smell and taste. You do this by checking the face that best represents your opinion on a scale with different faces with increasing satisfaction (see below as an example)..*


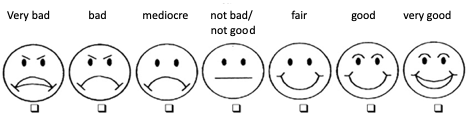


|  |  |  |  |  |  |  |
| --- | --- | --- | --- | --- | --- | --- |

*It is best to drink some water before you start tasting. You can do repeat this at any time during the tasting.*

*Please read carefully and be sure that you take the correct piece of hamburger as indicated on you questionnaire*

******Please take the conventional hamburger first /Please take the cultured hamburger first.*****

*Please examine the hamburger and anwers the following questions first before you start tasting*

- 1. How do you value the appearance of the hamburger?


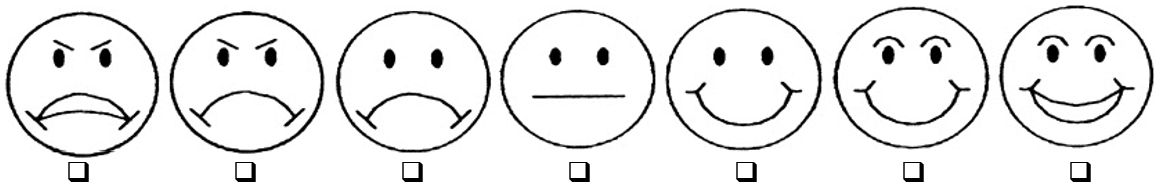


4.2 How is the color?


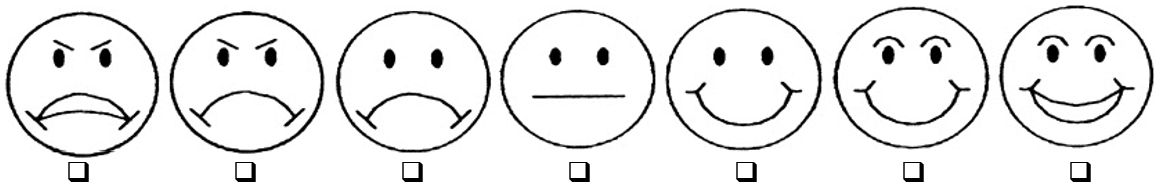


*Now, smell the hamburger and answer the following question* ***before*** *you start tasting !*

- 1. How is the smell?


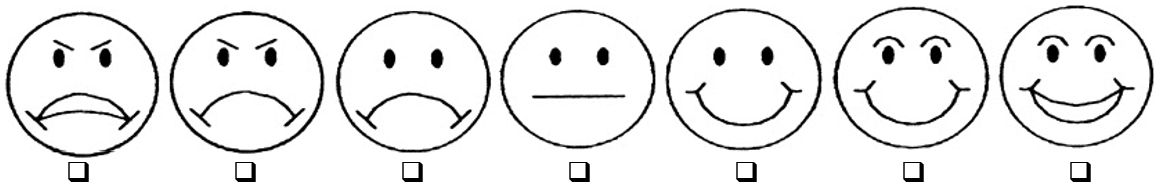


*Now you can taste the hamburger and answer the following questions*

- 1. how is the tenderness?


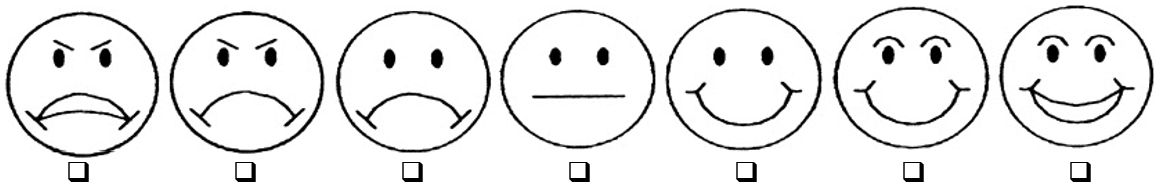


- 1. How is the juiciness?


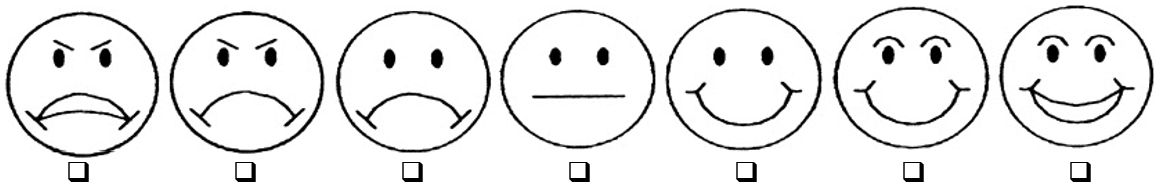


Now, please focus on the taste and evaluate it.

- 1. how does the hamburger taste?


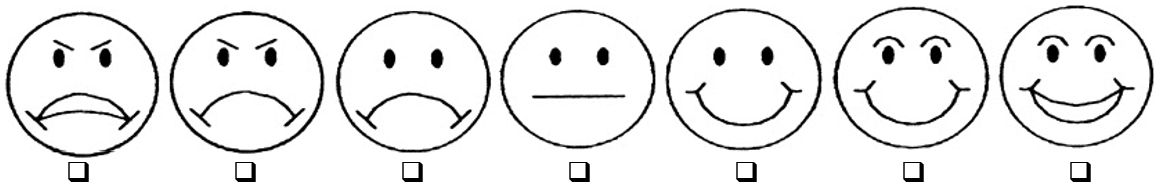


- 1. Do you experience an aftertaste?
- Yes
- No
  1. If yes, how was the aftertaste?
- Good
- Not good

*Please have a sip of water before you taste the next piece. Try to taste a same size piece as you did for the first one.*
(sequence is repeated for next piece of hamburger)


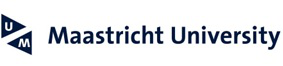
Q3

Ref:

**Study: Appreciation of meat by consumers**

*You are now ready with tasting and evaluation the hamburger. Please answer the following questions by checking the appropriate box (only 1 answer possible) or, when indicated, place your comments.*

- 1. What is your opinion on cultured meat?
- I am in favor
- I am probably in favor
- do not know
- I am probably against
- I am against
  1. Do you like to try cultured meat?
- definitely
- probably
- I do not know
- probably not
- definitely not
  1. Will you buy cultured meat when it becomes available in the supermarket?
- yes, definitely
- probably
- I do not know
- probably not
- definitely not

- 1. Are you willing to replace your current meat consumption by consumption of cultured meat?
- yes, definitely
- probably
- I do not know
- probably not
- definitely not
  1. Are you prepared to pay a premium for cultured meat?
- Yes
- No
  1. If yes, how much you will be willing to pay extra to enjoy the benefits of cultured meat? Consider the regular price for a hamburger to be € 1.
- € 1,10
- € 1,20
- € 1,50

€ 2,50

- € 5,00 or more

- 1. How do you feel about cultured meat? (thoughts, doubts, mixed feelings about benefits/down sides, additional value, risks, opportunities and so on)
     ………………………………………………………………………………………………………………………………………………………………………………………………………………………………………………………………………………………………………………………………………………………………………………………………………………………………………………………………………………

*Please answer the following questions about yourself.*

- 1. What is your highest level of education?
- vocational training
- lower professional education
- higher professonial education
- college or university education
- graduate education
  1. Wat is you current job status?
- paid employment
- self employed
- in between jobs
- unemployed
- housemaker
- student
- retired
- disabled
  1. Are you working in the meat industry (farm, slaughterhouse, meatpacker, butcher, BBQ restaurant or the like)
- Yes
- No
  1. What is your net household income?
- less than € 1.000
- € 1.000-€ 2.500
- € 2.500- € 4.000
- more than € 4.000


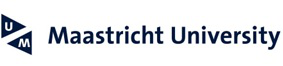
FQ

Ref:

**Study: Appreciation of meat by consumers**

Finally, last questions:

7 How comparable where the two hamburgers in your opinion ?

**Appearance?**

|  |  |  |  |  |  |  |  |  |  |  |
| --- | --- | --- | --- | --- | --- | --- | --- | --- | --- | --- |
| 0 | 1 | 2 | 3 | 4 | 5 | 6 | 7 | 8 | 9 | 10 |
| very different |  |  |  |  |  |  |  |  |  | the same |

**Smell?**

|  |  |  |  |  |  |  |  |  |  |  |
| --- | --- | --- | --- | --- | --- | --- | --- | --- | --- | --- |
| 0 | 1 | 2 | 3 | 4 | 5 | 6 | 7 | 8 | 9 | 10 |
| very different |  |  |  |  |  |  |  |  |  | the same |

**Taste?**

|  |  |  |  |  |  |  |  |  |  |  |
| --- | --- | --- | --- | --- | --- | --- | --- | --- | --- | --- |
| 0 | 1 | 2 | 3 | 4 | 5 | 6 | 7 | 8 | 9 | 10 |
| very different |  |  |  |  |  |  |  |  |  | the same |

Thank you for your participation! The investigator will join you for your payment and to debrief.
