# Supplementary material for: The effect of information content on acceptance of cultured meat in a tasting context
Source: PLoS One. 2020 Apr 16;15(4):e0231176. doi: 10.1371/journal.pone.0231176 (PMC7162467; doi:10.1371/journal.pone.0231176)
Supplement: Attachment — Submitted filename: Responses to reviewers.docx [file pone.0231176.s002.docx]

**Responses to comments made by reviewers and academic editor**

We appreciate the careful reading and thoughtful comments by the reviewers and made the necessary changes to the manuscript. Responses (R) point by point:

**Editor**

1. The manuscript was read and edited by a native Brit.
2. Please ensure that your manuscript meets PLOS ONE’s style requirements

R: We have made all the changes necessary to conform with the style requirements

3. Phrase “data not shown”, is not permitted.

R: Since the data are provided in detail through a repository (Mendeley), the data are actually retrievable, so we omitted the phrases “data not shown”.

4. Please ensure that you refer to Figure 2.

R: Apologies for this oversight. We now refer to figure 2.

5. Please remove any funding related text in the manuscript.

R: We removed the reference to funding. In the online submission, we have added the phrase on the role of funders in the study. These statements are also included in the cover letter

6. Competing interest section of online submission.

R: The statement that the Competing Interest has not altered adherence to the PLOS ONE policies on sharing data and materials has been included in the online submission and the cover letter.

7. Please include captions with supporting information.

R: We have added the supporting information to the Supplemental text at the bottom of the manuscript and provided a caption

**Reviewer 1**

P11. Effect of prior awareness on acceptance - reference [17] would show this more clearly than [11]. Next sentence, you have a crucial missing “not” - ‘was significant for subjects who had never heard of cultured meat or did not know exactly what it was, but NOT for those already knew…

R: The reference has been changed to the one suggested. Thanks for noting the missing ‘not’. We have added it.

P14. Solicited comments on cultured meat - the sentence here in parentheses should be complete - there is also a superfluous apostrophe here.

R: We have completed the sentence within parentheses. The quotation mark at the end of the parentheses is in our view correct as the entire phrase, including the part between parentheses, is part of the quote.

**Reviewer 2**

1. The authors discussed that the previous studies about culutured meat were conducted with students in agriculture and food sciences, and therefore the present study enployed a general population. In the Participants section, however, there is no information about participant’s faculty or profession. The authors should show information about participant’s faculty or profession in detail and the most of them do not major academic fields related to food sciences.

R:

2. In the experiment, the participants were provided the two samples of the same conventional meat hamburger with different labels, named “cultured” and “conventional” hamburgers. However, it seems that the authors did not check whether their experimental manipulation succeed, i.e. whether the participants did not aware that the two pieces of hamburgers were idential and not used cultured meat. If some participants had awared these deception procedures, the relaiability of the present results will become diminished. The authors should mention this problem.

R: This is a good point. We were aware at the beginning of the study that this could be a problem. In our pilot we made the two hamburger pieces exactly the same in size and shape and got the impression that participants did not believe them to be different. For that reason, we changed the size of the ‘cultured’ hamburger piece, which was not ideal for sensory analysis as this might have emphasized the exclusivity of the product. However, by changing the size, we have the strong impression that participants believed the nature of the ‘cultured’ hamburger, judged by their reactions during debriefing (one participant was so angry that he professed never to participate in a study at our University again). The issue of believing the ‘cultured’ nature of the sample was not formalized in a specific question, because we judged it ambiguous from an experimental point of view. Asking that question before debriefing, might have raised suspicion. After briefing, the question likely had resulted in difficult to interpret answers (e.g. not willing to admit that they were fooled).

We have added a paragraph in the discussion to mention this potential caveat.

Minor issues.
3. I think the contents of each information condition may be described not in the Appendix but in the main text of the paper because these are very important information in this study.

R: we have added the information boxes to the Methods section. This has inevitable increased the manuscript size with 512 words.

4. Figure 4. Please add the frequency or ratio (%) of each remark.

R: We have added the ratio of specific remarks per category to the figure 4.

5. The words “participants” and “subjects” are mixed in the manuscript. Please use the same expression throughout a manuscript.

R: we have replaced subjects by participants, except for the description of repeated measure analysis in within-subject and between-subject analyses. These descriptions were deemed to be standard statistical language.
